# Core self-evaluation and subjective wellbeing: A moderated mediation model

**DOI:** 10.3389/fpubh.2022.1036071

**Published:** 2022-12-20

**Authors:** Wei Chen, Tao Yang, Jing Luo

**Affiliations:** ^1^School of Psychology, Guizhou Normal University, Guiyang, China; ^2^Center for Big Data Research in Psychology, Guizhou Normal University, Guiyang, China; ^3^School of Education Science, Guizhou Education University, Guiyang, China

**Keywords:** core self-evaluation, subjective wellbeing, meaning in life, self-esteem, moderated mediation

## Abstract

**Objectives:**

Much has been written documenting the positive association between core self-evaluation and adolescents' subjective wellbeing, but little is known about the mediating and moderating mechanisms which underlay this relationship. This study constructed a moderated mediation model to examine whether meaning in life mediated the relationship between core self-evaluation and subjective wellbeing, and whether this mediating process was moderated by adolescents' self-esteem.

**Methods:**

A sample of 1,185 adolescents (11–17 years of age, 51.3% females) completed the Core Self-Evaluation Scale (CSES), the Meaning in Life Questionnaire (MLQ), the Rosenberg Self-esteem Scale (RSES), and the Index of Wellbeing Scale (IWS).

**Results:**

The results indicate that after controlling for gender and age, core self-evaluation contributed significantly to subjective wellbeing (β = 0.900, *p* < 0.001). Meaning in life played a mediating role in the relationship between core self-evaluation and subjective wellbeing (β = 0.143, *p* < 0.01), with core self-evaluation indirectly affecting subjective wellbeing through meaning in life (β_indirect_ = 0.068, 95% CI = [0.024, 0.119]). Self-esteem moderated the path mediated by meaning in life, more specifically, the conditional indirect effect between core self-evaluation and wellbeing was significant for adolescents with medium and low self-esteem (effect = 0.056, 95% CI = [0.014, 0.106]; effect = 0.092, 95% CI = [0.034, 0.159]. Both mediating and moderating effects were shown to exist in the association between core self-evaluation and adolescents' subjective wellbeing.

**Discussion:**

Based on the results, the following suggestions can be made: subjective wellbeing can be promoted through interventions such as enhancing adolescents' core self-evaluation and helping them understand the meaning in life, and that greater attention needs to be paid to adolescents with low self-esteem. The findings of this study helpful to clarify the mediation and moderating mechanism of the beneficial influence of adolescents' core self-evaluation on subjective wellbeing.

## Introduction

The value orientation of positive psychology is to turn the focus on mental diseases to the beautiful and positive aspects of human beings, help human beings to tap and stimulate internal qualities (1). As one of the three research focuses within positive psychology, subjective wellbeing has attracted much attention. Subjective wellbeing (SWB) is an individual's comprehensive judgment of overall quality of life based on self-defined standards (2), it is an important psychological indicator that reflects the quality of individual life (3). Adolescents are in a critical period of development, experiencing dramatic changes both physically and psychologically (4, 5). Studies have shown that SWB can not only maintain the mental health of adolescents, but also help improve adolescent academic performance (6–8). However, existing research on adolescents mainly focuses on problem behaviors such as internet addiction (9, 10), peer victimization (11, 12), suicidal ideation/behavior (13, 14), and have generally neglected positive development outcomes such as SWB. Mental health includes not only the physical absence of disease and maintaining a positive mental state, but also discovery and nurturing underlying positive forces (15). Wang and Ding (16) found that personality traits are closely related to subjective wellbeing. As the main influencing factor of subjective wellbeing, personality traits are the most effective predictor of subjective wellbeing (17).

Judge et al. (18) proposed four related personality traits: self-esteem, general self-efficacy, mental control source, and neuroticism, and they believed that these four aspects constitute the core self-evaluation of individuals. Core self-evaluation is a relatively stable personality trait, which refers to one's most basic evaluation and estimation of their self-ability and value (19). Individuals with high core self-evaluation tend to express themselves as having the ability to control their own lives (20) and experience less frustration in the face of negative events (21). It has been found that core self-evaluation is significantly related to job satisfaction (22, 23) and job performance (24, 25), and this kind of research mainly focuses on the related fields of industrial organization and management psychology. In recent years, research on core self-evaluation has gradually involved psychological fields such as mental health (26), depression (27), and psychological demands (28). Empirical research shows that core self-evaluation has a significant positive correlation with SWB (29) and can indeed predict one's level of SWB (30–32). Adolescents with low levels of core self-evaluation are likely to fall into a state of low levels of positive and negative emotions, leading to low life satisfaction (33). Guo et al. (34) took junior high school students as samples, indicating that core self-evaluation explained 15.1% of overall wellbeing, which can significantly positively predict overall life satisfaction, positive emotions and overall wellbeing. In secondary vocational students, core self-evaluation was significantly correlated with subjective wellbeing (*p* < 0.01), and could positively predict subjective wellbeing (β = 0.622, *p* < 0.01) (35). Nevertheless, less attention has been given to how and when core self-evaluation can improve SWB. Given the impact that core self-evaluation can have in guiding adolescents' positive development and preventing psychological and behavioral problems, the current study aimed to explore the influencing factors and formation mechanism of adolescents' SWB.

### The mediating role of meaning in life

Research into positive psychology has shown that sense of meaning in life plays an important role in individual psychological function (36). Meaning in life is a high-level psychological need, defined as one's understanding and experience of life and one's understanding of their life goals, missions, or tasks (37). Relevant studies have shown that individuals with higher core self-evaluation perceive more positive cognitions and emotions (21, 38), and research has shown that core self-evaluation has a predictive effect on meaning in life (39). Meaning in life is also closely related to personal mental health (40). A stronger sense of meaning in life has been shown to be related to positive experiences such as happiness, life satisfaction, and SWB (37, 41–44), and can predict one's level of psychological wellbeing (45). The more one perceives the meaning of life, the more one's positive emotions will be affected, in that they feel more happiness because they have clear life goals and concrete expectations for their future (46).

Overall, the current literature suggests that core self-evaluation is an important factor affecting both one's meaning in life and SWB, and that meaning in life can also affect SWB. This suggests that meaning in life is likely to mediate the relationship between core self-evaluation and SWB. Based on this, we established our first hypothesis:

Hypothesis 1: Meaning in life would (at least partially) mediate the relationship between core self-evaluation and adolescents' subjective wellbeing.

### The moderating role of self-esteem

Self-esteem is an individual's evaluation of self ability and value (47). As an emotional evaluation of one's overall self, self-esteem is an important moderating variable on the level of individual characteristics (48) and has generally been considered to be an important part of mental health (49, 50). One's level of self-esteem has been shown to be closely related to cognition, emotion, and behavior, and a high level of self-esteem can promote positive psychological development (51), influencing both one's pursuit of long-term goals and their positive attitudes toward self and happiness (52).

Terror management theory proposes that self-esteem is a kind of evaluation and sense of personal value, a feeling that one “is a valuable part of this meaningful world” (53), that is, an individual's experience of their sense of meaning in life and their intrinsic value. The meaning maintenance model suggests that self-esteem can maintain one's sense of meaning in life (54), and that to a certain extent, self-esteem can positively predict one's sense of life meaning (37)—namely the higher the level of self-esteem, the higher sense of life meaning (55). Individuals with a high level of self-esteem have a sense of control over the results of events in their lives, and tend to attribute these results to their efforts, therefore feeling they have strength and value, which then leads to them achieving a higher sense of life significance (56). Self-esteem is closely related to one's own development in that a stronger sense of self-esteem enhances one's perceived value of their own existence (57).

Studies have noted that self-esteem can be used as a predictor of SWB (58, 59). The stress-buffering model of high self-esteem shows that a high level of self-esteem helps individuals cope better with the negative consequences of bad events (60). Individuals with a high level of self-esteem use more problem-solving strategies when encountering adversity, usually hold a more positive attitude toward the future, and focus more on their own feelings rather than on social comparisons or expectations, therefore being less affected by the external world (61). In contrast, individuals with a low level of self-esteem choose to avoid and withdraw more when faced with negative events, and pay more attention to adverse consequences when failure occurs, which can easily lead to self-doubt (62). Furthermore, individuals with low self-esteem are more likely to feel stress and fear, resulting in lower levels of life satisfaction and positive emotions and higher negative emotions (63). The study found that self-esteem can significantly and positively predict individual life satisfaction (64, 65). High self-esteem is considered as a protective factor of life satisfaction, which can buffer the negative impact of stress on individual life satisfaction (66). Therefore, high self-esteem may be a protective factor in improving one's sense of meaning in life as well as their SWB, which leads to our second hypothesis:

Hypothesis 2: Self-esteem is the mediator between adolescents' sense of meaning in life and subjective wellbeing.

## Methods

### Participants and procedures

One thousand two hundred questionnaires were distributed across the nine middle schools. Fifteen participants did not fully complete the questionnaires, and after the elimination of these invalid questionnaires, the final sample was 1,185 adolescents (608 females; 51.3%). The effective rate of the questionnaire was 98.75%. The average age of participants was 13.60 ± 1.20 years old.

During the internship of psychology major students, using a convenience sampling method, a cross-sectional study was conducted with a sample made up of nine different middle schools from Guizhou Province in China. We printed the questionnaires used in the research into paper files, and researchers brought paper questionnaires to the schools contacted in advance. The class as a whole was used as a unit to carry out the group test. The author of this study and a trained graduate student majoring in psychology first obtained approval and cooperation from the teaching department of the school to use one period of the students' self-study time (~20 min). The aims and procedure of the study were explained to the students. After receiving participants' informed consent, participants completed the various questionnaires, which were collected at the end of the study period.

## Measures

### Core self-evaluation

The Core Self-Evaluation Scale (CSES) was compiled by Judge et al. (19), and the Chinese version of the CSES was translated by Zhang et al. (67). The scale is made up of 12 items which measure core self-evaluation, scored on a five-point Likert scale. A higher total score indicates a higher level of core self-evaluation. The Cronbach's α coefficient of the CSES in student groups by Yan (68) was 0.81, and the confirmatory factor analysis showed that the unidimensional models fit well. The Cronbach's α coefficient of the CSES in the study was 0.778.

### Meaning in life

The Meaning in Life Questionnaire (MLQ) was compiled by Steger et al. (37). This current study used the Chinese version of the MLQ, as developed by Chen et al. (69). There questionnaire uses 10 items to measure one's meaning in life, comprising two dimensions: presence of meaning and search of meaning. Each item is ranked using a seven-point Likert scale, and a higher total score indicates a higher sense of meaning in life. The MLQ showed good reliability among middle school students (Cronbach's α coefficient = 0.736) and stable factor structure in the study of Chen et al. (69). In the current study, the Cronbach's α coefficient of the MLQ was 0.728.

### Self-esteem

The Rosenberg Self-Esteem Scale (RSES) is a 10-items self-report questionnaire used to assess self-esteem (70). The current study used the Chinese version of the RSES as revised by Wang et al. (71). Each item is ranked using a four-point Likert scale. A higher total score indicates a higher level of individual self-esteem. In previous studies, the Cronbach's α coefficient of the RSES in Chinese middle school students was 0.87, respectively, and confirmatory factor analysis showed that the model was well fitted (72). The Cronbach's α coefficient of the RSES in the current study was 0.794.

### Subjective wellbeing

The Index of wellbeing Scale (IWS) measures an individual's level of SWB (64). The current study used Li and Zhao (73) Chinese version of the IWS. The scale consists of two parts: the overall emotion index and life satisfaction. The former consists of eight items, while the latter has only one item. Each item is scored on a seven-point Likert scale. To calculate the total score, the average score of the overall scale is added to the score of the life satisfaction items with a weight of 1.1. A higher score indicates a higher level of SWB. The Cronbach's α coefficient of the IWS among Chinese students was 0.866 (69). In the current study, the Cronbach's α coefficient of the scale was 0.932.

### Statistical analysis

EpiData version 3.1 was used to input data and SPSS version 25.0 was used for statistical analysis. Following recommended procedure (74), Harman's single-factor test was carried out to test the common method variance bias. Next, descriptive analysis, correlation analysis, and regression analysis were used to analyze the study variables. The hypothetical model was analyzed using the PROCESS macro for SPSS provided by Hayes (75). The SPSS macro PROCESS (Model 4) was used to test whether the relationship between core self-evaluation and SWB was mediated by meaning in life (Hypothesis 1). We used the SPSS macro PROCESS (Model 14, a moderated mediation model) to test whether the mediation process was regulated by self-esteem (Hypothesis 2). Five thousand bootstrap samples were selected to obtain 95% bias-corrected confidence intervals (CIs). If the 95% CI does not contain zero, this suggests that the indirect effects are significant (76).

## Results

### Common method deviation

The research variables were included in the exploratory factor analysis using SPSS 25.0 software, and the unrotated principal component analysis was carried out on all 41 measured items according to the Harman single factor test (74). The results showed that there were seven common factors with eigenvalues greater than one, with the first factor explaining 21.23% of the variation, which is less than the critical standard of 40% (77). Therefore, the data of this study was not affected by the deviation of the common method.

### Descriptive statistics and correlation analysis

Table 1 presents the means, standard deviations, and Pearson correlation coefficients for core self-evaluation, meaning in life, self-esteem, and SWB. Correlation analysis showed that adolescents' core self-evaluation was positively correlated with meaning in life (*r* = 0.291, *p* < 0.001), self-esteem (*r* = 0.646, *p* < 0.001), and SWB (*r* = 0.366, *p* < 0.001); meaning in life was significantly positively correlated with self-esteem (*r* = 0.30, *p* < 0.001) and SWB (*r* = 0.185, *p* < 0.001); self-esteem was significantly positively correlated with SWB (*r* = 0.339, *p* < 0.001). The results of correlation analysis provided preliminary evidence for the hypotheses.

**Table 1:** Descriptive statistics and correlation analysis (*N* = 1,185).

**Variables**	**M (SD)**	**1**	**2**	**3**	**4**	**5**	**6**
1. Gender	1.51 (0.50)	–					
2. Age	13.60 (1.20)	−0.046	–				
3. Core self-evaluation	3.06 (0.61)	−0.138^**^	−0.157^***^	–			
4. Meaning in life	4.76 (0.95)	−0.025	0.017	0.291^***^	–		
5. Self-esteem	2.76 (0.50)	−0.100^**^	−0.089^**^	0.646^***^	0.300^***^	–	
6. SWB	4.75 (1.50)	−0.014	−0.113^***^	0.366^***^	0.185^***^	0.339^***^	–

Gender: 1 = males, 2 = females.

** *p* < 0.01,

*** *p* < 0.001.

### Testing for mediation

PROCESS macro Model 4 (75) was used to test the mediating effect of meaning in life on the relationship between core self-evaluation and SWB (Hypothesis 1). The results of the mediation model are shown in Table 2.

**Table 2:** Testing the mediation effect of meaning in life on SWB.

**Predictors**	**Model 1 (SWB)**		**Model 2 (Meaning in life)**		**Model 3 (SWB)**	
	**β**	***t***	**β**	***t***	**β**	***t***
Gender	0.101	1.240	0.038	0.714	0.096	1.178
Age	−0.069	−1.997*	0.051	2.309^*^	−0.076	−2.214^*^
Core self-evaluation	0.900	13.103^***^	0.474	10.681^***^	0.832	11.615^***^
Meaning in life					0.143	3.190^**^
*R* ^2^	0.138		0.089		0.146	
*F*	63.232^***^		38.399^***^		50.336^***^	

^*^Indicates significant paths:

* *p* < 0.05,

** *p* < 0.01,

*** *p* < 0.001.

As shown in Table 2 (Model 1), after controlling for covariates, core self-evaluation positively predicted SWB (β = 0.900, *p* < 0.001). In Model 2, core self-evaluation positively predicted meaning in life (β = 0.474, *p* < 0.001). In Model 3, when meaning in life was entered as a mediator, it positively predicted SWB (β = 0.143, *p* < 0.01), while the direct effect of core self-evaluation on SWB was substantially reduced (β = 0.832, *p* < 0.001). Bootstrapping analysis showed that the indirect effects of core self-evaluation on SWB (β_indirect_ = 0.068, 95% CI = [0.024, 0.119]) through meaning in life was significant, indicating that a mediation model had been established. Indirect effects accounted for 7.536% of the total effects. In summary, results showed that Hypothesis 1 was supported. The relationship between core self-evaluation and SWB was partially mediated by meaning in life. It is worth noting that the role of the mediation was relatively small, and as such, these results need to be interpreted with caution.

### Testing for moderated mediation

**Figure 1:**
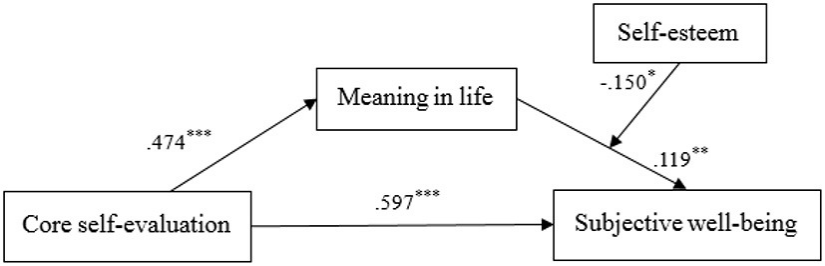
Path coefficients of the moderated mediation model. ^*^Indicates significant paths: ^*^p < 0.05, ^**^p < 0.01, ^***^p < 0.001.

SPSS PROCESS macro Model 14 (75) was used to test the moderating effect of self-esteem (Hypothesis 2) on the mediation model. Table 3 and Figure 1 show the results of the moderated mediation model. When the interaction between meaning in life and self-esteem were significant and the 95% CI did not contain zero, the conditional indirect effect was held. As shown in Figure 1 and Table 4, self-esteem significantly moderated the indirect effect of core self-evaluation on SWB through meaning in life. This finding showed that meaning in life was positively associated with SWB (β = 0. 119, *p* < 0.01), and the product (interaction item) of meaning in life and self-esteem had a significant impact on SWB (β = −0.150, *p* < 0.05). Furthermore, the direct effect of core self-evaluation on SWB was 0.597 (*p* < 0.001).

**Table 3:** Results of self-esteem moderate the mediation process.

**Predictors**	**Model 1 (Meaning in life)**		**Model 2 (SWB)**	
	**β**	***t***	**β**	***t***
Gender	0.038	0.714	0.104	1.293
Age	0.051	2.309^*^	−0.076	−2.233^*^
Core self-evaluation	0.474	10.681^***^	0.597	6.707^***^
Meaning in life			0.119	2.638^**^
Self-esteem			0.490	4.651^***^
Meaning in life × Self-esteem			−0.150	−2.011^*^
*R* ^2^	0.089		0.164	
*F*	38.399^***^		38.473^***^	

^*^Indicates significant paths:

* *p* < 0.05,

** *p* < 0.01,

*** *p* < 0.001.

**Table 4:** Conditional indirect effect of self-esteem when meaning in life mediated between core self-evaluation and SWB.

**Mediator**	**Self-esteem**	**Effect**	**BootSE**	**95% CI**
Meaning in life	−1 SD	0.092	0.032	[0.034, 0.159]
	M	0.056	0.023	[0.014, 0.106]
	+1 SD	0.021	0.029	[−0.035, 0.079]

**Figure 2:**
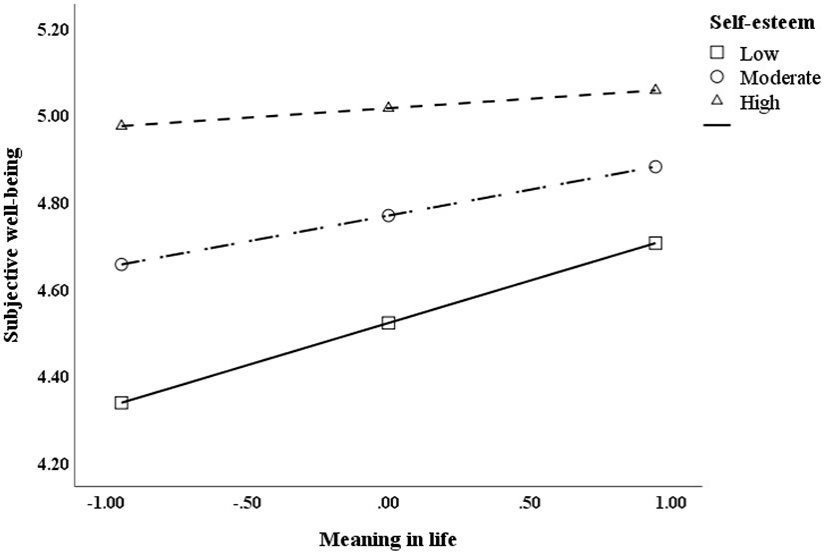
Simple slopes.

To further clarify the mediating role of self-esteem in obtaining meaning in life and SWB, self-esteem was grouped by adding or subtracting a standard deviation from the mean, and a simple slope test was conducted to examine the relationship between meaning in life and SWB at different levels of self-esteem (Figure 2). The results showed that when the level of self-esteem was high, meaning in life could not significantly predict SWB (bsimple = 0.044, *t* = 0.775, *p* > 0.05). However, when self-esteem was at a medium or low level, meaning in life significantly predicted SWB (bsimple = 0.119, *t* = 2.638, *p* < 0.01; bsimple = 0.195, *t* = 3.183, *p* < 0.01). Bias-corrected percentile bootstrap analysis further showed that core self-evaluation indirectly affected SWB through meaning in life, and was moderated by self-esteem. More specifically, the conditional indirect effect between core self-evaluation and SWB was significant for adolescents with medium and low self-esteem (effect = 0.056, 95% CI = [0.014, 0.106]; effect = 0.092, 95% CI = [0.034, 0.159]), see Table 4. In summary, these results indicate that self-esteem moderated the relationship between core self-evaluation and SWB through meaning in life, partially supporting Hypothesis 2.

## Discussion

Although the influence of core self-evaluation on adolescents' SWB has been confirmed by numerous of studies (29–31, 78), little was known about its potential mediators (i.e., how core self-evaluation works) and regulatory mechanisms (i.e., when core self-evaluation works). To address this gap in understanding, the current study introduced two key variables—meaning in life and self-esteem—and constructed and tested a moderated mediation model. Hypothesis 1 was supported by the conclusion that core self-evaluation had a direct influence on SWB, and meaning in life was an important intermediary factor. Hypothesis 2 was partially supported, adolescents' self-esteem can moderating meaning in life, thereby affecting SWB.

### Hypothesis 1: The mediating role of meaning in life

This study found that core self-evaluation positively predicted meaning in life, which was consistent with previous studies (39). The higher the individual's core self-evaluation, the more self-affirmation and identification they exhibited, and the more positively they were able to experience and seek out meaning in life, meaning the higher their level of meaning in life. Meaning in life positively predicted SWB, which was consistent with previous research (41, 42), that is, the stronger one's sense of meaning in life, the easier it is for them to gain a sense of happiness. Furthermore, core self-evaluation promoted adolescents' SWB through their sense of meaning in life. As a personality trait, core self-evaluation has a direct effect on external information processing through cognitive schemas (79). “Cognitive schemas” is a cognitive structure formed by people in the process of understanding the world around them (80). The development of cognitive schemas is the result of the interaction of “assimilation” and “adaptation”, the cognitive subject enriches his cognitive structure by incorporating new external stimuli into his original cognitive schema through the role of “Assimilation”; through the accumulation of “Adaptation”, it causes the adjustment and change of the original cognitive structure, gradually adapts to the new stimulus, and realizes the improvement of cognitive level. Research shows that individuals with low core self-evaluation will experience more boredom and easily lose track of current goals (81). The middle school students with low core self-evaluation view themselves in a negative perspective, lack sufficient confidence to solve problems when encountering interpersonal relationships or academic pressure, and often fall into repeated thinking rather than taking action (82), difficulty getting a sense of achievement from life and poor life experience. If this situation continues, it is difficult for individuals to realize the benign development of cognitive schemas, leading to a reduction in their perception of their own value and purpose of existence, thus reducing their SWB. In contrast, individuals with a high core self-evaluation are not so easily influenced by the outside world (83), allowing them to have a more positive outlook, to recognize their value, and to meet the high-level psychological needs of adolescents (i.e., meaning in life). Therefore, meaning in life is shown to provide positive emotional experiences for adolescents. Students with high sense of meaning in life have clearer goals in life and more solid expectations for the future, enabling them to feel more happiness.

### Hypotheses 2: The moderating roles of self-esteem

The results of this study show that adolescents' self-esteem moderates the indirect effect between core self-evaluation and SWB. Compared to students with a higher level of self-esteem, the indirect effect was more significant in adolescents with a lower level of self-esteem, which implies that improving an individual's self-esteem can increase one's sense of meaning in life. The specific moderating point lies in the second half of the intermediary chain, in that the relationship between meaning in life and SWB depends on adolescents' self-esteem level. It should be pointed out that this moderated mode does not mean that self-esteem is a risk/disadvantage factor of SWB. As shown in Figure 2, the SWB of individuals with a high level of self-esteem was generally higher than SWB of individuals with a low level of self-esteem. The reason for this moderation may be that the SWB of individuals with high self-esteem was already at a higher level, so there was less possibility for meaning in life to have an impact; however for those with a low level of self-esteem, the increase of meaning in life had an opportunity to have a stronger impact in increasing their SWB (54). As the existing literature demonstrates Individuals with a high level of self-esteem tend to think that the outcome of circumstances depends primarily on factors within their own control. As they have a sense of control over the outcome, they make more effort to achieve their goal and are able to use more appropriate coping strategies in the face of setbacks and trauma (84). Their sense of control over events makes them more likely to feel strength and value, and feel higher life significance (85). In contrast, individuals with low self-esteem tend to think that the results of events are difficult to control and as such, generally have a lower sense of self-identity (86). Thus, they are more likely to feel helpless when facing pressure and are not usually willing to make much effort to deal with pressures (87), making it more difficult for them to feel a sense of value and meaning in their lives. The improvement of self-esteem is usually a relatively slow process. People's subjective wellbeing can be improved by increasing positive emotions, enhancing the sense of control over events, and helping experience and understand the meaning of life. Bad family environment (88, 89), stressful life events and the destruction of established interpersonal relationships (90) will have a negative impact on individual self-development and cause low self-esteem. The attachment theory of self-esteem believes that a good family environment can provide individuals with all kinds of resources necessary for growth, and then develop high self-esteem (89). Schools and parents can guide adolescents to make appropriate attribution, and give them more opportunities to exercise their abilities in order to continuously acquire self-efficacy. In addition, appropriate teacher support, peer support, and opportunities for self-determination about the child, academics, and school affairs can help improve self-esteem in children and adolescents (90).

### Limitations

There are however some limitations in the current study. First, the study participants were junior high school students and the sample size was small, also limits due to cultural context, making it uncertain whether our results can be extended to any students. Future research should expand the scope of participants and test the results in other countries or regions. Second, this study used a cross-sectional design, which cannot accurately infer the causal relationship between variables. Future research should use a tracking design to further test the findings of the current study.

## Conclusion

The results of the current study support the following: first, core self-evaluation has a significant positive predictive effect on adolescents' SWB; second, meaning in life plays a mediating role in the relationship between core self-evaluation and SWB, in that core self-evaluation indirectly affects SWB through meaning in life; finally, self-esteem moderates the mediated path through meaning in life, such that this indirect effect is much stronger for adolescents with low self-esteem relative to those with high self-esteem.

In brief, the findings of our study show that enhancing the level of meaning in life and self-esteem can promote the SWB of adolescents. It is helpful to clarify the mediation and moderating mechanism of the beneficial influence of adolescents' core self-evaluation on SWB, and has certain theoretical significance and practical value in improving adolescents' SWB. Our findings provide some practical application. People's SWB can be improved by increasing positive emotions, enhancing their sense of control over events, and helping them experience and understand the meaning in life.

## Data availability statement

The original contributions presented in the study are included in the article/supplementary material, further inquiries can be directed to the corresponding author.

## Ethics statement

The studies involving human participants were reviewed and approved by Committee of the School of Psychology of Guizhou Normal University. Written informed consent to participate in this study was provided by the participants' legal guardian/next of kin.

## Author contributions

WC concepted the article and provided framework of the manuscript. TY analyzed the data and drafted the manuscript. The final version was approved by WC and JL. All authors contributed to the article and approved the submitted version.
